# A chromosome-scale *Mytilus edulis* genome assembly for aquaculture, marine ecology, and evolution

**DOI:** 10.1093/g3journal/jkae138

**Published:** 2024-06-27

**Authors:** Tim Regan, Tiago S Hori, Tim P Bean

**Affiliations:** The Roslin Institute and Royal (Dick) School of Veterinary Studies, University of Edinburgh, Midlothian, EH25 9RG, UK; Atlantic Aqua Farms Ltd., Charlottetown, Prince Edward Island, PE C1A 4A2, Canada; The Roslin Institute and Royal (Dick) School of Veterinary Studies, University of Edinburgh, Midlothian, EH25 9RG, UK

**Keywords:** aquaculture, evolution, bivalve, mussel, *Mytilus edulis*, *Mytilidae*

## Abstract

The smooth-shelled blue mussel, *Mytilus edulis* is part of the *Mytilus* species complex, encompassing at least three putative species: *M. edulis, Mytilus galloprovincialis*, and *Mytilus trossulus*. These three species occur on both sides of the Atlantic and hybridize in nature, and both *M. edulis* and *M. galloprovincialis* are important aquaculture species. They are also invasive species in many parts of the world. Here, we present a chromosome-level assembly of *M. edulis*. We used a combination of PacBio sequencing and Dovetail's Omni-C technology to generate an assembly with 14 long scaffolds containing 94% of the predicted length of the *M. edulis* genome (1.6 out of 1.7 Gb). Assembly statistics were as follows: total length = 1.65 Gb, N50 = 116 Mb, L50 = 7, and L90 = 13. BUSCO analysis showed 92.55% eukaryote BUSCOs identified. AB-*Initio* annotation using RNA-seq from mantle, gills, muscle, and foot predicted 47,128 genes. These gene models were combined with IsoSeq validation resulting in 45,379 full CDS protein sequences and 129,708 isoforms. Using GBS and shotgun sequencing, we also sequenced several eastern Canadian populations of *Mytilus* to characterize single-nucleotide as well as structural variance. This high-quality genome for *M. edulis* provides a platform to develop tools that can be used in breeding, molecular ecology and evolution to address questions of both commercial and environmental perspectives.

## Introduction

**Fig. 1.**
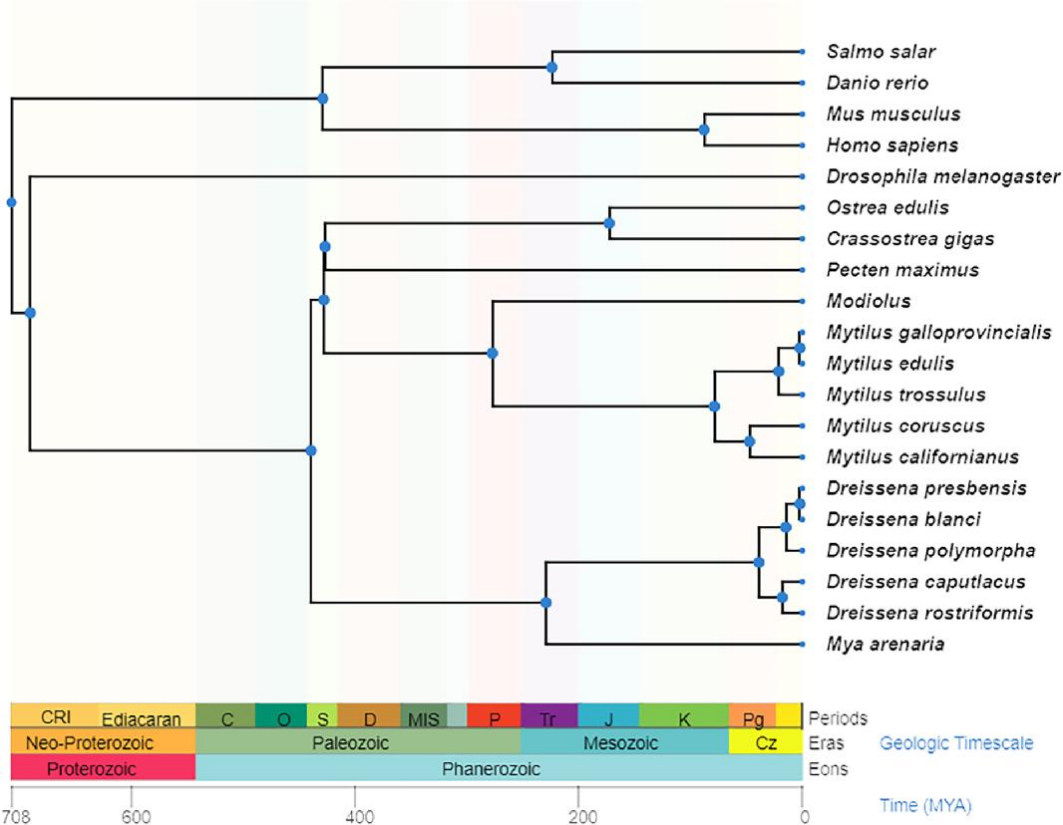
TimeTree for *M. edulis* mytilidae diverged ∼387 MYA, Mytilus diverged ∼78 MYA and the *M. edulis* species complex diverged ∼20 MYA. Generated using TimeTree (Kumar *et al*. 2022).

The smooth-shelled blue mussel (*Mytilus edulis*) is common to the North Atlantic from Arctic to Mediterranean regions, with habitat ranging from upper shore to the shallow subtidal (Hayward and Ryland 2017). *M. edulis* is known to hybridize with *Mytilus trossulus* in North America and with *M. trossulus* and *Mytilus galloprovincialis* in Europe. Together, these three species form what is referred to as the *Mytilus edulis* species complex (Fig. 1) (McDonald *et al*. 1991). However, these species are known to also hybridize with putative *Mytilus* species in the southern hemisphere: *Mytilus chilensis* (Chile), *Mytilus platensis* (Argentina and Uruguay), *Mytilus aoteanus* (New Zealand) and *Mytilus planulatus* (Australia) (Oyarzún *et al*. 2021). Collectively, it is more appropriate to refer to them as the *Mytilus* spp. complex.

This reef-building bivalve is an ecosystem engineer. *Mytilus* spp. dominate fouling communities in shallow and substrata providing important secondary habitat (Norling and Kautsky 2007). Offshore wind energy structure surveys found that they can cover the structures with up to 3.4 kg of biomass m^−2^ (Krone *et al*. 2013). Through filter-feeding, eutrophication is reduced which can alter ecosystems (Broszeit *et al*. 2016). This nutrient cycling ability has been harnessed by using *M. edulis* to study the fate of persistent organic and metal pollutants (Chase *et al*. 2001; McEneff *et al*. 2014), for the bioremediation of waste (Broszeit *et al*. 2016) and reduce environmental effects from salmon farms (MacDonald *et al*. 2011).

Blue mussels (*Mytilus* spp.), a key bivalve production species (FAO 2020), face decreasing wild spat availability for aquaculture in the UK and elsewhere (Regan *et al*. 2021). These losses are attributed to multiple stressors including warmer seas (Seuront *et al*. 2019) causing a poleward range contraction (Jones *et al*. 2010). Additionally, warmer oceans elevate dissolved CO_2_ leading to Ocean Acidification (OA) impacting mussel viability (Asplund *et al*. 2014) and disease resistance (Ellis *et al*. 2015). This increases the susceptibility of mussels to bacterial pathogenesis (Ripabelli *et al*. 1999; Eggermont *et al*. 2017), with emerging pathogens representing a persistent threat (Charles *et al*. 2020; Cano *et al*. 2022). Infectious disease such as disseminated Neoplasia (DN) of *M. trossulus* origin is associated with reduced fitness in *Mytilus* spp. (Burioli *et al*. 2021). Furthermore, the effects of hybridization between the species of the *Mytilus* spp. complex in the northern hemisphere are yet uncertain with suggested negative effect of *M. trossulus* hybridization and potential adaptive introgression in the case of *M. galloprovincialis* hybridization (Fraïsse *et al*. 2014; Kenchington *et al*. 2020; Michalek *et al*. 2021).

To protect the aquaculture industry from these threats, hatchery efforts have been launched in the UK (Regan *et al*. 2021), elsewhere in Europe (Kamermans *et al*. 2013) and in Canada (Gurney-Smith *et al*. 2017). However, these efforts have not been straightforward, and a better understanding of fundamental biology is required to achieve commercial success. Despite their importance in aquaculture and the valuable ecosystem services they provide, no chromosomal assembly existed for any species within the *Mytilus* species complex prior to this study in 2021. Improved genomic tools are required to address fundamental biological questions such as inheritance patterns and adaptations.

Like many bivalves, the mussel genome is highly heterozygous (3.5%) with an estimated 43% repeat content (Corrochano-Fraile *et al*. 2022). The linear plot of k-mer abundance analysis clearly shows a heterozygous peak in addition to the homozygous peak and estimates a shorter haploid genome length of 1.18 Gb compared with 1.71 G estimate from flow-cytometry (Rodríguez-Juíz *et al*. 1996). These characteristics make assembly of these genomes challenging. However, recently genomes for the American oyster, Pacific Oyster, *Mytilus corruscus* and *Mytilus californianus* have been assembled to chromosome-level using short and long-read sequencing technologies as well as Hi-C-based scaffolding (Yang *et al*. 2021; Paggeot *et al*. 2022a; Gomez-Chiarri *et al*. 2015; Penaloza *et al*. 2021). We used a similar approach in this project to produce a highly contiguous assembly. Practical application of this assembly is demonstrated in cross-species synteny analyses and in population structure of *Mytilus* individuals sampled from different regions of the Canadian Atlantic.

## Methods

### DNA extraction, library preparation for genome assembly

One naïve (unsexed) *M. edulis* sample (named “Anne”) was selected from samples collected by the Provincial Department of Communities and Fisheries in the estuary Foxley river in Prince Edward Island (PEI). Foxley River was selected as a sampling site because there is no grow-out aquaculture there (i.e. seed from other bays are not transferred to the area). Due to potential introgression of other species of the *Mytlius* species complex (e.g. *M. trossulus*), this sample was genotyped using 12 SNPs described by (Wilson *et al*. 2018). We sampled the foot, gill, mantle, hemolymph, and muscle of sample “Anne” aseptically, flash froze fresh tissues in liquid nitrogen and preserved them at −80°C. Tissues were shipped to Dovetail Genomics in Scott's Valley, CA, in excess dry ice. Dovetail extracted high molecular weight (HMW) DNA from adductor muscle tissue using an in-house modified CTAB method. Dovetail prepared and sequenced PacBIO SMRTbell libraries with SPRI bead size selection of 30 kb + to a depth of 196 × using the Sequel II sequencer producing ∼15 million PacBio CLR reads (∼340 Gb). Processed PacBio data (from Dovetail, SMRT LINK V11) can be found on SRA under accession number SRX11246493.

### Raw contig assembly, scaffold formation, and polishing

Dovetail generated a primary contig-level assembly using wtdbg2 (Ruan and Li 2020). This primary assembly was 1.96 Gb long in 17,825 contigs and a N50 of 443 Kb. The contig-level assembly was filtered of putative duplicated haplotypes and contaminants using Purge Haplotigs (Roach *et al*. 2018) and Blobtools2 (Challis *et al*. 2020), respectively. Non-molluscan contigs were removed according to Blobtools results. After haplotype purging and contaminant removal, the final contig assembly had 10,111 contigs, for a total of 1.65 Gb and a N50 of 518 Kb. Scaffolding was performed using Omni-C libraries of muscle tissue (SRA Accession SRR28966249) sequenced using Illumina HiSeq 2000 and the HiRise assembler (v1.0) to generate a primary chromosome-level assembly made of 2,117 scaffolds. The same DNA sample used by Dovetail was shipped to UWM (University of Wisconsin—Madison) and sequenced to a 100 × using the NovaSeq 2000 sequencer (SRR28968509). These data were trimmed using Trimmomatic (v0.39) and used for polishing with racon (v1.4.3) (Vaser *et al*. 2017). This code is available in the Github repository https://github.com/timregan/M.-edulis-assembly. This final assembly was further filtered to contain only sequences > 5,000 bp. The resulting draft is deposited on NCBI assembly under accession number GCA_019925275.1. The final assembly is made of 1,119 scaffolds and has an N50 of 116 Mb. The 14 putative *M. edulis* chromosomes are deposited under accession numbers CM034349.1 to CM34362.1.

Completeness was evaluated using compleasm v0.2.5 (Huang and Li 2023) (eukaryota_odb10: 255 BUSCOS, metazoa_odb10: 954 BUSCOS and mollusca_odb10: 5295 BUSCOS) (Manni *et al*. 2021) and Merqury (Rhie *et al*. 2020). Merqury analysis was carried out with the same read set used for polishing as the original PacBio CLR reads were not suitable for this analysis. General length metrics were obtained using QUAST (v5.0.1) (Gurevich *et al*. 2013; Mikheenko *et al*. 2018). Synteny mapping between our *M. edulis* assembly and the *M. trossulus* (GCF_036588685.1, Pacific Northwest Research Institute) was done using the MCScanX.h function of MCScanX (v2) (Wang *et al*. 2012). Putative orthologous groups were identified with Orthofinder (v.2.5.4) using predicted gene structures for *M. edulis* (this work) and *M. trossulus*. Dot plots and circle plots were generated using MCScanX (v.2). We generated circular plots using CIRCOS (v.0.69-9) and the MScanX collinearity file.

### RNA preparation and IsoSeq analysis

Circular Consensus Sequencing (CCS) data was generated from gill, muscle, hemolymph, and foot, which was analyzed using IsoSeq3. We shipped flash-frozen gill and adductor muscle tissue from sample “Anne” to the Biotechnology Centre Core facility at the University of Wisconsin, Madison (UWM). Samples were homogenized using a Qiagen Tissuelyser (2 min @ 20 Hz). RNA was extracted using the RNeasy Mini Kit (Qiagen) with on-column DNAse treatment. UWM performed RNA QC with a nanodrop and bioanalyzer and prepared libraries using the Iso-seq Library SMRTbell express template prep kit. Libraries were sequenced using one Sequel II SMRT cell in CCS mode (i.e. HiFi reads). UWM provided de-multiplexed processed HiFi reads for gill (1.3 million reads—∼3.9 Gbs) and muscle (1.6 million reads—∼4.9 Gbp) (Gill—SRR29044552; Muscle—SRR29044553). RNA samples from foot, mantle, gill, hemolymph and muscle were also sent to the Genome Excellence Centre (Genome Quebec) in Montreal. Additional HiFi sequencing was carried out as above for hemolymph (1.7 million reads—∼5.2 Gbp and foot (2.03 million reads—∼7.4 Gbp) (SRA Accession Numbers SRR28976541 to SRR28976543). Using gill, mantle, muscle and foot samples four stranded pair-end libraries were made and sequenced in an Illumina HiSeq2000 (SRA accession numbers SRR28976540 to SRR28976543). Putative full-length (FL) transcripts were identified using the IsoSeq3 pipeline (https://github.com/ylipacbio/IsoSeq3). Putative open read frames (ORFs) were identified using TransDecoder (v5.5.0) (https://github.com/TransDecoder/TransDecoder/wiki).

### Annotation

Repeat modeler (4.1.0) (Flynn *et al*. 2020) was used to predict repeat motifs for *M. edulis* and Repeat Masker (2.0.2a) (Smit *et al*. 2015) was used to mask the final assembly (Supplementary Table 1). Ab-initio annotation was done using Augustus (v3.4.0) (Stanke *et al*. 2006) trained with genes from *M. galloprovincialis*, *M. coruscus,* and *Crassostrea virginica*. Additional Augustus “hints” were generated using short-read RNA-seq (gill, foot, muscle, and mantle) and IsoSeq data generated herein. The ab-initio annotation was updated using PASA (v2.5) with alignments of a de-novo transcriptome produced using Trinity (v2.8.15) (Grabherr *et al*. 2011) in Genome-Guided mode. Two runs through PASA were used to update the *ab-initio* annotation. FL transcripts from IsoSeq3 pipeline were mapped to the genome using pbmm2 with pre-sets for IsoSeq and filtered based on quality IsoSeq3 collapse (minimum alignment identity/coverage: 0.90/0.90) and IsoSeq3 refine. We used the resulting gff annotation to run SQANTI3 (v4.3.0). We used sqanti_qc.py to generate quality data for sqanti_filter.py. Filtering to remove artifacts was carried out using the default parameters. Finally, ab-initio predictions and filtered IsoSeq FLs were merged with AGAT (v.1.2.0). Using agat_sp_merge.pl, we removed duplicate gene models/isoform and assigned orphan isoforms from the IsoSeq data when possible. Amino acid sequences were translated from the CDS using agat_sp_extract_sequences using options -t “CDS” -p –cfs –acs –asc.

### Sample collection for SNP discovery and population structure

**Fig. 2.**
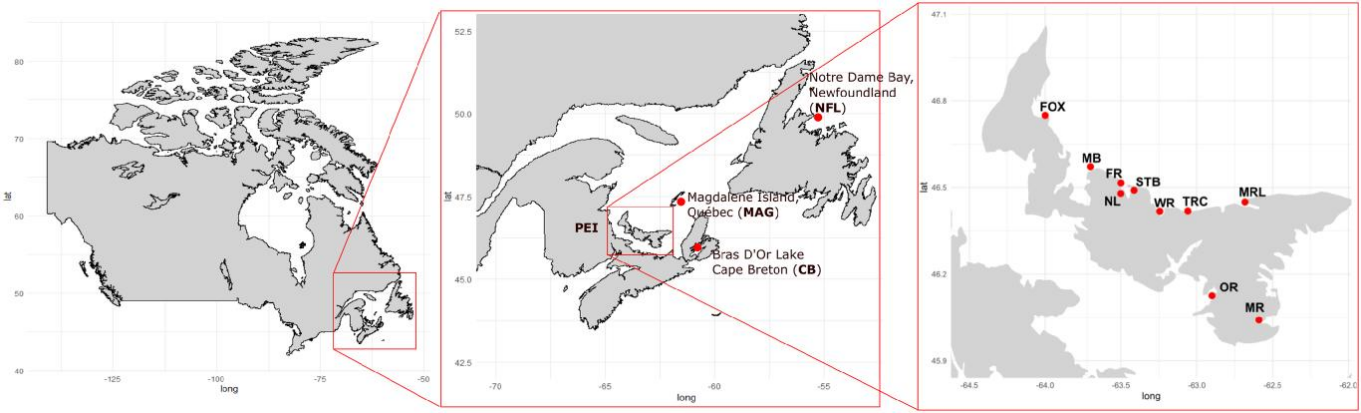
Sampling locations in PEI for population genetics analysis, mussels were sampled in sets of 96 from the Bras D'or Lake in Cape Breton (Nova Scotia), the Magdalene Island (Québec) and Notre Dame Bay in Newfoundland. Samples were collected from several regions across PEI, labeled from west to east: Foxley River (FOX) (wild population), Malpeque Bay (MB) (North Shore, Prince County); French River (FR), New London (NL), Stanley Bridge (STB), Whetley River (WR), Tracadie (TRC) (North Shore, Queen county); Orwell (OR) (South Shore, Queen's county; Morell (MRL) (North Shore—King's county) and; Murray River (MR) (South Shore, King county).

We collected gill samples for DNA extractions using standard molecular biology techniques and preserved them in non-denatured 100% ethanol. For population genetics analysis, we sampled mussels in sets of 96 from west to east PEI in Foxley River (FOX) (wild population), Malpeque Bay (MB) (North Shore, Prince County); French River (FR), Stanley Bridge (ST), Whetley River (WR), Tracadie (TRC) (North Shore, Queen county); Orwell (OR) (South Shore, Queen's county; Morell (MRL) (North Shore—King's county) and; Murray River (MR) (South Shore, King county) (Fig. 2). Seed deployed on these sites were originally collected in St. Peter's bay, Brudenell River, and MB. We also collected samples in the Bras D'or Lake in Cape Breton (Nova Scotia), the Magdalene Island (Québec) and Notre Dame bay in Newfoundland.

### SNP markers, admixture analysis, population structures

Samples for SNP discovery and population genetics were shipped to LGC genomics in Berlin. Restriction-associated DNA libraries (RAD) libraries were prepared by LGC with using restriction enzyme MslI, normalized and Illumina sequenced using a NextSeq 2000 sequencer. The resulting reads were trimmed and checked for the restriction site by LGC. This final read set was used to identify SNPs and call individual genotypes using Tassel5 (v.5.2.4). SNPs were filtered by Minor Allele Frequency (MAF) < 0.01, depth (75 > DP < 500), and % of individuals with genotypes (90%). For the SNP discovery, the samples from Cape Breton were excluded from the filtering analysis. These samples were shown to be a pure *M. trossulus* population and had missing calls for a significant number of sites. For population genetics analysis, a second set of SNPs (MAF > 0.05, 10 > DP < 100, only bi-allelic sites with genotypes called in 95% of samples—4,610 SNPs) that were successfully called across all populations was used. Allele frequency distribution and the deviation of observed from expected heterozygosity frequencies (based on HW equilibrium) are shown in Supplementary Fig. 1. The observed heterozygosity levels are not greater than expected levels over the vast majority of the genome, indicating no large levels of selection or drift, as expected for an unbiased set of SNPs. All samples were also genotyped for the 12 SNPs from (Wilson *et al*. 2018) used to discriminate between *M*. edulis, *M. trossulus* and *M. galloprovincialis*.

**Fig. 3.**
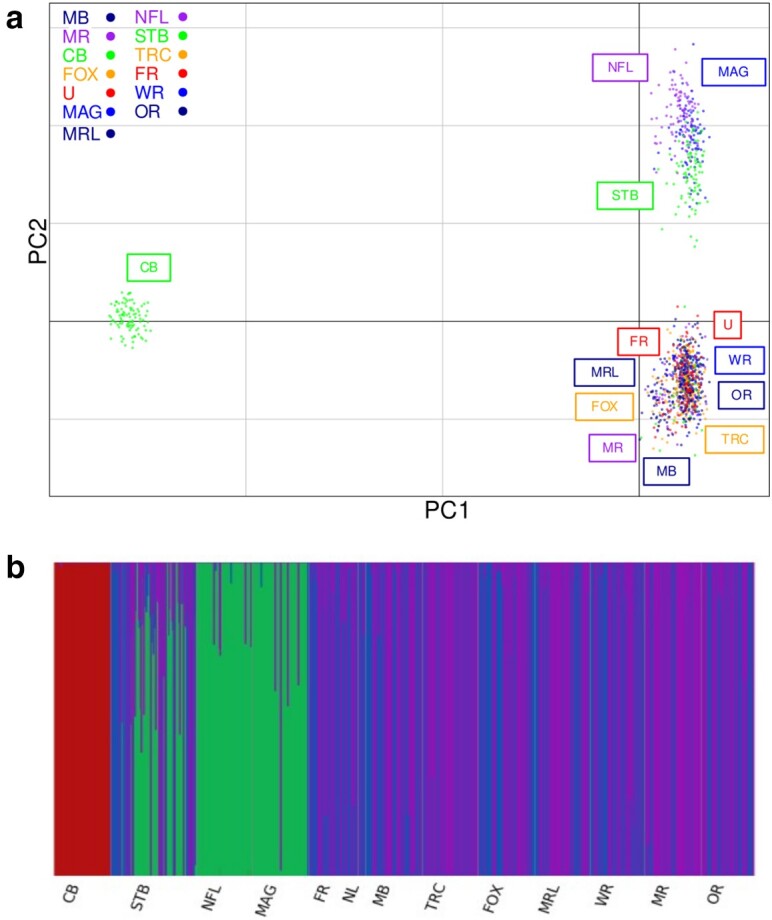
Blue mussel (*Mytilus* spp.) population structure in the North American Atlantic. a) PCA with 4,610 SNPs, b) fastStructure with 4,610 SNPs. Samples are from Cape Breton, Nova Scotia (CB); Magdalene Islands, Quebec (MAG) and Notre Dame Bay, Newfoundland (NFL); PEI samples are from French River (FR), Malpeque Bay (MB), New London (NL), Tracadie (TRC), Foxley River (FOX) (wild population), Morell (MRL), Whetley River (WR), Murray River (MR) and Orwell (OR) and Stanley Bridge (STB).

Principal Component Analysis (PCA) (Fig. 3a) was performed using the pca.dudi function of the R package Adegenet (v.2.1.7). FastSTRUCTURE (v1.0) (K 1 to K12, –cv == 10) was used to analyze the genome-wide SNP data from herein. Model complexity was determined using the chooseK.py script provided with fastSTRUCTURE (Fig. 3b). STRUCTURE (LOCDATA = 1 NOADMIX = 0, LINKAGE = 0, USEPOPINFO = 1, LOCPRIOR = 0, INFERALPHA = 1, FPRIORMEAN = 0.01, FPRIORSD = 0.05) was used to analyze the species discrimination SNPs (12 markers from Wilson *et al*. 2018) and included the genotypes published by (Wilson *et al*. 2018) as outgroups (data not shown).

## Results and discussion

### Genome assembly and annotation

The chromosome-level assembly presented herein was produced in two stages. First, PacBio CLR reads (∼340 Gb) were produced, representing coverage of 196 × for an estimated genome size of 1.7 Gb (Hinegardner 1974; Rodríguez-Juíz *et al*. 1996). These reads were assembled into contigs using wtdbg2, which uses uncorrected reads (Ruan and Li 2020). The primary assembly was 1.96 Gb long in 17,825 contigs and a N50 of 443 Kb. After haplotype purging and contaminant removal, the final contig assembly had 10,111 contigs, for a total of 1,65 Gb and a N50 of 518 Kb. Following scaffolding using Omni-C libraries and the HiRise assembler, we generated a primary chromosome-level assembly made of 2,117 scaffolds. This assembly was further filtered to contain only sequences > 5,000 bp. The resulting draft is deposited on NCBI assembly under accession number GCA_019925275.1. The final assembly is made of 1,119 scaffolds and has an N50 of 116 Mb. The 14 putative *M. edulis* chromosomes are deposited under accession numbers CM034349 to CM34362. Detailed statistics for the assemblies can be found in Table 1.

**Table 1.** Summary statistics for *M. edulis* species complex assemblies from this study (GCA_019925275.1), *M. edulis* from the Darwin Tree of Life (GCA_963676685.2), *M. trossulus* from the Pacific Northwest Research Institute (GCA_036588685.1), and *M. galloprovincialis* from Yantai University (GCA_037788925.1).

	Anne (This study)	xbMytEdul2.2, Darwin Tree of Life (2024)	PNRI_Mtr1.1.1.hap1, Pacific Northwest Research Institute (2024)	*MytGallo_primary_0.1*, Yantai University (2024)
***Species***	*M. edulis*	*M. edulis*	*M. trossulus*	*M. galloprovincialis*
***Genome size (Gb)***	1.7	1.4	1.3	1.4
***# of scaffolds***	1,119	2,368	540	94
***# of contigs***	9,866	3,557	1,371	425
***Scaffold N50 (Mb)***	116.5	93.4	85.4	96.9
***Coverage***	196.0x	30.0x	45.0	38.0x
***QV***	32.39	NA	NA	NA
***Completeness (Merqury)***	76.71	NA	NA	NA
***BUSCO mollusca_odb10, n:5295***	Complete: 88.5% Single copy: 77.2%, Duplicated: 11.3%, Fragmented: 1.2%, Missing: 10.3%	NA	Complete:96.1% Single copy: 89.8%, Duplicated: 6.3%, Fragmented: 0.2%, Missing:3.7%	NA

Compared to the three most recent chromosome-level assemblies available on NCBI, the assembly presented herein has better contiguity as measured by scaffold N50 (Table 1). Both *M. edulis* assemblies’ length falls close to the estimated size of the genome based on c-value (1.7) (Rodríguez-Juíz *et al*. 1996) and is significantly longer than what is estimated by k-mer abundance analysis with GenomeScope (1.18 Gb) (Supplementary Fig. 2).

Despite the total assembly length being close to that estimated using c-values, k-mer-based completeness analysis recovers only 76% in a set of Illumina reads origination from sample “Anne”. When the putative purged haplotigs were added back to the assembly, recovery was ∼83%. This apparent low k-mer recovery is likely due to the error rate in the original PacBio data. However, other possible explanations are the consensus being different from other haploid genomes, the fact that the reads used for polishing were not used from the primary assembly, the contigs removed based on length or contaminant status, and the gaps arising from the Omni-C scaffolding. It is also possible that the high heterozygosity affects the accuracy of k-mer abundance analysis, as shown by the large discrepancy between genome size estimates.

Completeness analysis in Merqury resulted in 76.71% recovery of k-mers present in the polishing Illumina data from the final version of the assembly. We also evaluated the completeness of the assembly when combined with purged haplotypes, which was 83.44%. QV value for the primary assembly was 32.39, while the combined draft had a QV of 30.73. While Merqury relies on k-mer-based analyses between raw reads and assembly, completeness was also estimated using compleasm which relies on a BUSCO (Benchmarking Universal Single-Copy Orthologs) database. Compleasm BUSCO analysis showed 92.55%, 91.95%, and 88.5% complete BUSCOS against the eukaryote, metazoan and molluscan databases, respectively. The 14 putative chromosomes represent ∼96% of the assembly, with lengths varying from 140 Mbp to 90 Mbp. These data and the N50 metric show that this assembly has high contiguity and that this assembly and its annotation will be highly useful for aquaculture, evolution and molecular ecology studies. Herein, we illustrate the possible applications of this assembly by performing population and synteny analyses.

### SNP discovery and population structure

Due to the close relationship between the members of the *Mytilus* species complex, we wanted to verify that the individual sampled (Anne) was pure *M. edulis*. We genotyped 895 PEI individuals, 96 samples from Magdalene Island and 96 individuals from Newfoundland of the same 12 SNP panel. We also included 96 individuals from Cape Breton (NS), which has long been considered a pure *M. trossulus* population. We generated two sets of SNPs from the genotyping-by-sequencing (GBS) data: the first set totaling 71,231 SNPs using only samples from PEI and made a polymorphic collection of SNPs in *M. edulis.* The second set, with 4,610 markers, is a polymorphic set of SNPs in both *M. edulis* and *M. trossulus.* Genotypes for both marker sets were called in 1,183 samples above using GBS.

In the PCA, untrained clustering clearly separated both CB from PEI/NL/MAG and also the putative populations in the Gulf of St. Lawrence. PCA and fastSTRUCTURE analysis indicates that there is low population stratification between different regions of PEI, while other off-island populations display more separation. PCA and fastSTRUCTURE also indicate that there is no introgression of Cape Breton Genetics (i.e. *M. trossulus*) in PEI. Therefore, we are confident that the sample “Anne” represents an *M. edulis* individual. However, we only genotyped 96 samples collected in an area with no grow-out leases (Foxley River—FOX) while all other samples came from areas with the presence of mussel aquaculture. Although unlikely, we cannot rule out the possibility of minor introgression of *M. trossulus* genetics masked by a sampling bias in aquaculture sites favoring *M. edulis.* Population structure and putative admixture are shown in Fig. 3.

### Annotation and synteny analysis

**Fig. 4.**
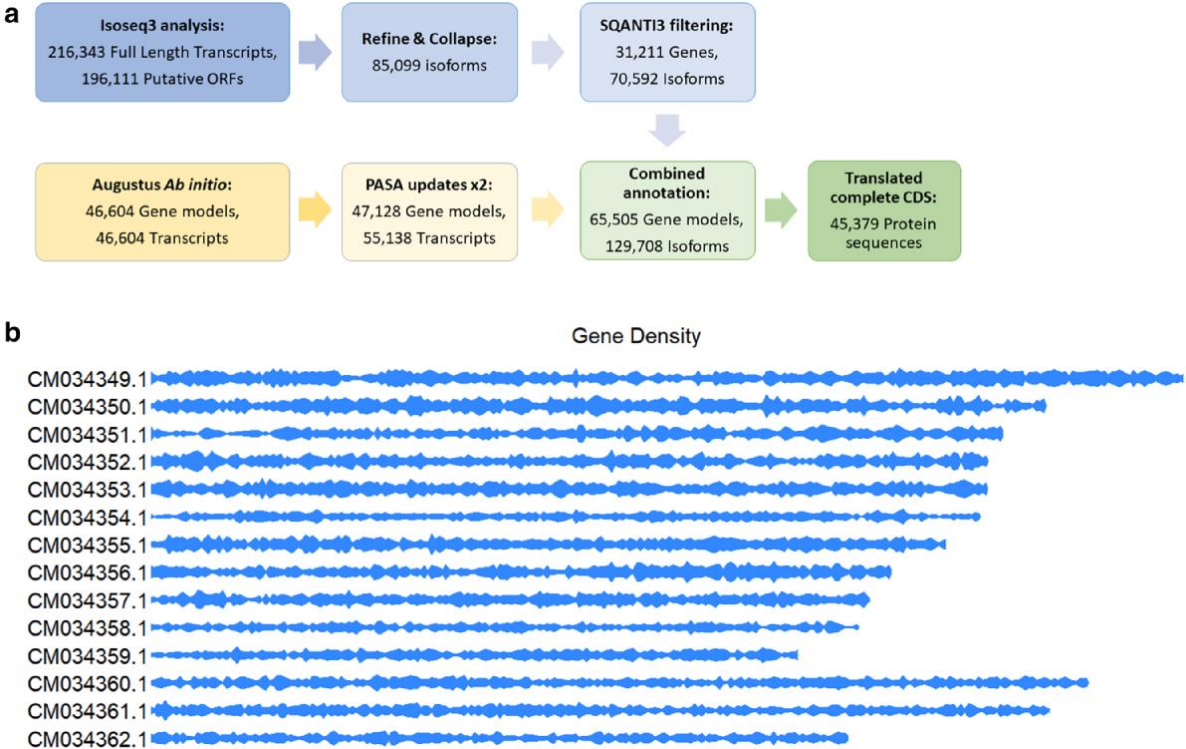
Annotation schematic and gene density. a) A schematic of the annotation pipeline used and (b) the density of genes along each of the chromosomes (made using R package karyoploteR).

*Ab-initio* gene prediction in Augustus detected 46,604 gene models that produced 46,604 transcripts after filtering based on evidence support. After two rounds of PASA updates, the final number of gene models was 47,128 and the number of transcripts was 55,138. Using IsoSeq3 analysis of CCS data from muscle and gill (2.9 million reads, 188,165 Mb), we identified 196,111 putative open reading frames from the 216,343 FL transcripts. BLASTp analysis against the uniref90 database returned informative hits for ∼80% (164,969) of these translated transcripts. After IsoSeq refine and collapse 85,099 isoforms survived, and following SQANTI3 filtering, 70,592 isoforms remained. They were assigned to 31,211 unique genes. Finally, these models were combined with the Augustus/PASA analyses to yield 65,505 gene models and 129,708 isoforms. Proteins were translated from the CDS ensuring only complete CDS were translated and that isoforms were not incorrectly fused together. This resulted in 45,379 amino acid sequences. The annotation pipeline and density of the final 45,379 protein sequences along each of the chromosomes is displayed in Fig. 4. Compleasm BUSCO analysis of these 45,379 proteins showed a recovery of 78.43 and 76.83% complete BUSCOS against the eukaryote and metazoan databases, respectively.

**Fig. 5.**
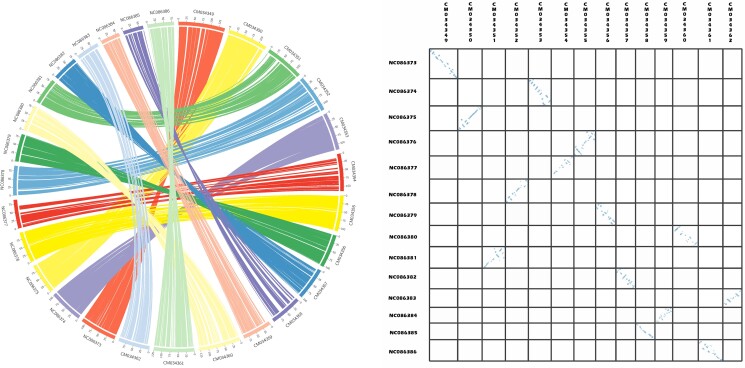
Synteny between the *M. edulis* and *M. trossulus* putative chromosomes. Syntenic orthologous regions between the *M. edulis* and *M. trossulus* genome assemblies are displayed by Circos plot (left) and dot plot (right).

Synteny analysis showed a high degree of collinearity between putative chromosomes of *M. edulis* and *M. trossulus* (Fig. 5). However, putative inversions, transpositions and deletions can be observed in almost all chromosomes. Gene order in chromosomes represented by sequences CM034349 (*M. edulis* chromosome 1)—NC086373 (*M. trossulus* chromosome 1) and CM034353 (*M. edulis* chromosome 5)—NC086374 (*M. trossulus* chromosome 2) showed the highest conservation. Putative orthologous relationships between *M. edulis* and *M. trossulus* chromosomes are shown in Table 2.

**Table 2.** Putative synteny between *M. edulis* and *M. trossulus*.

*M. edulis*	*M. trossulus*
Chromosome 1 (CM034349.1)	Chromosome 1 (NC_086373.1)
Chromosome 2 (CM034350.1)	Chromosome 3 (NC_086375.1)
Chromosome 3 (CM034351.1)	Chromosome 9 (NC_086381.1)
Chromosome 4 (CM034352.1)	Chromosome 6 (NC_086378.1)
Chromosome 5 (CM034353.1)	Chromosome 2 (NC_086374.1)
Chromosome 6 (CM034354.1)	Chromosome 5 (NC_086377.1)
Chromosome 7 (CM034355.1)	Chromosome 4 (NC_086376.1)
Chromosome 8 (CM034356.1)	Chromosome 7 (NC_086379.1)
Chromosome 9 (CM034357.1)	Chromosome 10 (NC_086382.1)
Chromosome 10 (CM034358.1)	Chromosome 13 (NC_086385.1)
Chromosome 11 (CM034359.1)	Chromosome 12 (NC_086384.1)
Chromosome 12 (CM034360.1)	Chromosome 8 (NC_086380.1)
Chromosome 13 (CM034361.1)	Chromosome 14 (NC_086386.1)
Chromosome 14 (CM034362.1)	Chromosome 11 (NC_086383.1)

Ids are NCBI Assembly database molecule name and accession number.

The taxonomic status of the “species” in the *Mytilus* species complex remains in debate. Chromosome-level assemblies allow the study of macroevolution of the genome by looking at synteny across species. Herein, we present the synteny analysis between the 14 putative chromosomes of *M. edulis* and *M. trossulus* to exemplify how chromosome-level assemblies may allow us to better understand the phylogenetic relationships within the genus *Mytilus*. This analysis shows that some of putative orthologous chromosomes of the 2 species maintain high levels of collinearity (e.g. chromosomes 1 and 5 from *M. edulis* with 1 and 2 from *M. trossulus*, respectively) while others present significant levels of re-arrangements (e.g. chromosomes 7 and 11 from *M. edulis* with chromosomes 4 and 12 from *M. trossulus* respectively). Future studies providing an in-depth analysis of chromosome synteny will shed light on the level of collinearity between multiple members of the genus *Mytilus*. Homology between chromosomes is a key element of the viability of hybrids. Reproductive isolation tends to increase during speciation (Mayr 1947), and these resources will permit further studies on the reproductive compatibility of the species in genus *Mytilus* at the chromosome level.

Here, we present a highly contiguous chromosome assembly for *Mytilus edulis* confirming species-level individual purity through resequencing. To date, our resource has been applied in multiple studies analyzing *Mytilus* genome assemblies (Paggeot *et al*. 2022; Gallardo-Escarate *et al*. 2023) and cross-species gene orthology analyses (Saco *et al*. 2023a). Recently, the concept of a pangenome for *M. galloprovincialis* has received increased attention (Gerdol *et al*. 2020) with “core genes” and “dispensable genes” being described across populations of *M. galloprovincialis (*Saco *et al*. 2023b*)*. Future studies investigating hybridization across *Mytilus* spp. will be able to investigate pangenome structure across *Mytilus* spp. with highly contiguous assemblies such as our own. The gene annotations produced in this study were generated using Augustus gene model predictions integrating full transcript IsoSeq data and applying stringent filtering parameters. This comprehensive approach provides a robust foundation for future cross-species analyses and biological studies on gene function within the *Mytilus* species complex.

## Supplementary Material

jkae138_Supplementary_Data

## Data Availability

The resulting draft is deposited on NCBI assembly under accession number GCA_019925275.1. Raw genomic sequencing data is available at NCBI BioProject PRJNA740305. Code used for assembly is described in https://github.com/timregan/M.-edulis-assembly. Genome annotation and translated protein sequences are available on Figshare 10.6084/m9.figshare.25101872. Supplemental material available at G3 online.

## References

[jkae138-B1] Asplund ME , Baden SP, Russ S, Ellis RP, Gong N, Hernroth BE. 2014. Ocean acidification and host-pathogen interactions: blue mussels, Mytilus edulis, encountering Vibrio tubiashii. Environ Microbiol. 16(4):1029–1039. doi:10.1111/1462-2920.12307. 24147969

[jkae138-B2] Broszeit S , Hattam C, Beaumont N. 2016. Bioremediation of waste under ocean acidification: reviewing the role of Mytilus edulis. Mar Pollut Bull. 103(1–2):5–14. doi:10.1016/j.marpolbul.2015.12.040. 26778338

[jkae138-B3] Burioli EAV , Hammel M, Bierne N, Thomas F, Houssin M, Destoumieux-Garzón D, Charrière GM. 2021. Traits of a mussel transmissible cancer are reminiscent of a parasitic life style. Sci Rep. 11(1):24110. doi:10.1038/s41598-021-03598-w. 34916573 PMC8677744

[jkae138-B4] Cano I , Parker A, Ward GM, Green M, Ross S, Bignell J, Daumich C, Kerr R, Feist SW, Batista FM. 2022. First detection of Francisella halioticida infecting a wild population of blue mussels Mytilus edulis in the United Kingdom. Pathogens. 11(3):329. doi:10.3390/pathogens11030329. 35335653 PMC8953295

[jkae138-B5] Challis R , Richards E, Rajan J, Cochrane G, Blaxter M. 2020. BlobToolKit—interactive quality assessment of genome assemblies. G3 (Bethesda). 10(4):1361–1374. doi:10.1534/g3.119.400908. 32071071 PMC7144090

[jkae138-B6] Charles M , Villalba A, Meyer G, Trancart S, Lagy C, Bernard I, Houssin M. 2020. First detection of Francisella halioticida in mussels Mytilus spp. Experiencing mortalities in France. Dis Aquat Organ. 140:203–208. doi:10.3354/dao03505. 32815528

[jkae138-B7] Chase ME , Jones SH, Hennigar P, Sowles J, Harding GCH, Freeman K, Wells PG, Krahforst C, Coombs K, Crawford R, et al 2001. Gulfwatch: monitoring spatial and temporal patterns of trace metal and organic contaminants in the Gulf of Maine (1991–1997) with the blue mussel, Mytilus edulis L. Mar Pollut Bull. 42(6):490–504. doi:10.1016/S0025-326X(00)00193-4. 11468927

[jkae138-B8] Corrochano-Fraile A , Davie A, Carboni S, Bekaert M. 2022. Evidence of multiple genome duplication events in Mytilus evolution. BMC Genomics. 23(1):340. doi:10.1186/s12864-022-08575-9. 35501689 PMC9063065

[jkae138-B9] Eggermont M , Bossier P, Pande GSJ, Delahaut V, Rayhan AM, Gupta N, Islam SS, Yumo E, Nevejan N, Sorgeloos P, et al 2017. Isolation of Vibrionaceae from wild blue mussel (Mytilus edulis) adults and their impact on blue mussel larviculture. FEMS Microbiol Ecol. 93:4. doi:10.1093/femsec/fix039. 28334251

[jkae138-B10] Ellis RP , Widdicombe S, Parry H, Hutchinson TH, Spicer JI. 2015. Pathogenic challenge reveals immune trade-off in mussels exposed to reduced seawater pH and increased temperature. J Exp Mar Biol Ecol. 462:83–89. doi:10.1016/j.jembe.2014.10.015.

[jkae138-B11] FAO . 2020. The state of world fisheries and aquaculture 2020. Sustainability in action. The State of World Fisheries and Aquaculture (SOFIA). Rome, Italy: FAO. p. #244.

[jkae138-B12] Flynn JM , Hubley R, Goubert C, Rosen J, Clark AG, Feschotte C, Smit AF. 2020. RepeatModeler2 for automated genomic discovery of transposable element families. Proc Natl Acad Sci USA. 117(17):9451–9457. doi:10.1073/pnas.1921046117. 32300014 PMC7196820

[jkae138-B13] Fraïsse C , Roux C, Welch JJ, Bierne N. 2014. Gene-flow in a mosaic hybrid zone: is local introgression adaptive? Genetics. 197(3):939–951. doi:10.1534/genetics.114.161380. 24788603 PMC4096372

[jkae138-B14] Gallardo-Escárate C , Valenzuela-Muñoz V, Nuñez-Acuña G, Valenzuela-Miranda D, Tapia FJ, Yévenes M, Gajardo G, Toro JE, Oyarzún PA, Arriagada G, et al 2023. Chromosome-Level genome assembly of the blue mussel Mytilus chilensis reveals molecular signatures facing the marine environment. Genes (Basel). 14(4):876. doi:10.3390/genes14040876. 37107634 PMC10137854

[jkae138-B15] Gerdol M , Moreira R, Cruz F, Gómez-Garrido J, Vlasova A, Rosani U, Venier P, Naranjo-Ortiz MA, Murgarella M, Greco S, et al 2020. Massive gene presence-absence variation shapes an open pan-genome in the Mediterranean mussel. Genome Biol. 21(1):275. doi:10.1186/s13059-020-02180-3. 33168033 PMC7653742

[jkae138-B16] Gomez-Chiarri M , Warren WC, Guo X, Proestou D. 2015. Developing tools for the study of molluscan immunity: the sequencing of the genome of the eastern oyster, Crassostrea virginica. Fish Shellfish Immunol. 46(1):2–4. doi:10.1016/j.fsi.2015.05.004. 25982405

[jkae138-B17] Grabherr MG , Haas BJ, Yassour M, Levin JZ, Thompson DA, Amit I, Adiconis X, Fan L, Raychowdhury R, Zeng Q, et al 2011. Full-length transcriptome assembly from RNA-Seq data without a reference genome. Nat Biotechnol. 29(7):644–652. doi:10.1038/nbt.1883. 21572440 PMC3571712

[jkae138-B18] Gurevich A , Saveliev V, Vyahhi N, Tesler G. 2013. QUAST: quality assessment tool for genome assemblies. Bioinformatics. 29(8):1072–1075. doi:10.1093/bioinformatics/btt086. 23422339 PMC3624806

[jkae138-B19] Gurney-Smith HJ , Wade AJ, Abbott CL. 2017. Species composition and genetic diversity of farmed mussels in British Columbia, Canada. Aquaculture. 466:33–40. doi:10.1016/j.aquaculture.2016.08.038.

[jkae138-B20] Hayward PJ , Ryland JS. 2017. Handbook of the Marine Fauna of North-West Europe. Oxford: Oxford University Press.

[jkae138-B21] Hinegardner R . 1974. Cellular DNA content of the Mollusca. Comp Biochem Physiol A Comp Physiol. 47(2):447–460. doi:10.1016/0300-9629(74)90008-5. 4156206

[jkae138-B22] Huang N , Li H. 2023. Compleasm: a faster and more accurate reimplementation of BUSCO. Bioinformatics. 39(10):btad595. doi:10.1093/bioinformatics/btad595. 37758247 PMC10558035

[jkae138-B23] Jones SJ , Lima FP, Wethey DS. 2010. Rising environmental temperatures and biogeography: poleward range contraction of the blue mussel, Mytilus edulis L., in the western Atlantic. J Biogeogr. 37(12):2243–2259. doi:10.1111/j.1365-2699.2010.02386.x.

[jkae138-B24] Kamermans P , Galley T, Boudry P, Fuentes J, Mccombie H, dos Reis Batista IC, Blanco Garcia A, Dominguez L, Cornette F, Pincot L. 2013. 11—blue Mussel hatchery technology in Europe. In: Allan G, Burnell G, editors. Advances in Aquaculture Hatchery Technology. Sawston, Cambridge: Woodhead Publishing. p. 339–373.

[jkae138-B25] Kenchington EL , MacDonald BW, Cogswell A, Hamilton LC, Diz AP. 2020. Sex-specific effects of hybridization on reproductive fitness in Mytilus. J Zool Syst Evol Res. 58(2):581–597. doi:10.1111/jzs.12348.

[jkae138-B26] Krone R , Gutow L, Joschko TJ, Schröder A. 2013. Epifauna dynamics at an offshore foundation—implications of future wind power farming in the North Sea. Mar Environ Res. 85:1–12. doi:10.1016/j.marenvres.2012.12.004. 23312860

[jkae138-B27] Kumar S , Suleski M, Craig JM, Kasprowicz AE, Sanderford M, Li M, Stecher G, Hedges SB. 2022. TimeTree 5: an expanded resource for Species divergence times. Mol Biol Evol. 39(8):msac174. doi:10.1093/molbev/msac174. 35932227 PMC9400175

[jkae138-B28] MacDonald BA , Robinson SMC, Barrington KA. 2011. Feeding activity of mussels (Mytilus edulis) held in the field at an integrated multi-trophic aquaculture (IMTA) site (Salmo salar) and exposed to fish food in the laboratory. Aquaculture. 314(1):244–251. doi:10.1016/j.aquaculture.2011.01.045.

[jkae138-B29] Manni M , Berkeley MR, Seppey M, Zdobnov EM. 2021. BUSCO: assessing genomic data quality and beyond. Current Protocols. 1(12):e323. doi:10.1002/cpz1.323. 34936221

[jkae138-B30] Mayr E . 1947. Ecological factors in speciation. Evolution. 1(4):263–288. doi:10.2307/2405327.

[jkae138-B31] McDonald J , Seed R, Koehn R. 1991. Allozymes and morphometric characters of three species ofMytilus in the Northern and Southern Hemispheres. Mar Biol. 111(3):323–333. doi:10.1007/BF01319403.

[jkae138-B32] McEneff G , Barron L, Kelleher B, Paull B, Quinn B. 2014. A year-long study of the spatial occurrence and relative distribution of pharmaceutical residues in sewage effluent, receiving marine waters and marine bivalves. Sci Total Environ. 476:317–326. doi:10.1016/j.scitotenv.2013.12.123. 24472720

[jkae138-B33] Michalek K , Vendrami DLJ, Bekaert M, Green DH, Last KS, Telesca L, Wilding TA, Hoffman JI. 2021. Mytilus trossulus introgression and consequences for shell traits in longline cultivated mussels. Evol Appl. 14(7):1830–1843. doi:10.1111/eva.13245. 34295367 PMC8288009

[jkae138-B34] Mikheenko A , Prjibelski A, Saveliev V, Antipov D, Gurevich A. 2018. Versatile genome assembly evaluation with QUAST-LG. Bioinformatics. 34(13):i142–i150. doi:10.1093/bioinformatics/bty266. 29949969 PMC6022658

[jkae138-B35] Norling P , Kautsky N. 2007. Structural and functional effects of Mytilus edulis on diversity of associated species and ecosystem functioning. Mar Ecol Prog Ser. 351:163–175. doi:10.3354/meps07033.

[jkae138-B36] Oyarzún PA , Toro JE, Nuñez JJ, Suárez-Villota EY, Gardner JPA. 2021. Blue mussels of the Mytilus edulis species complex from South America: the application of species delimitation models to DNA sequence variation. PLoS One. 16(9):e0256961. doi:10.1371/journal.pone.0256961. 34473778 PMC8412288

[jkae138-B37] Paggeot LX , DeBiasse MB, Escalona M, Fairbairn C, Marimuthu MPA, Nguyen O, Sahasrabudhe R, Dawson MN. 2022. Reference genome for the California ribbed mussel, Mytilus californianus, an ecosystem engineer. J Hered. 113(6):681–688. doi:10.1093/jhered/esac041. 35947871 PMC9710001

[jkae138-B38] Penaloza C , Gutierrez AP, Eory L, Wang S, Guo X, Archibald AL, Bean TP, Houston RD. 2021. A chromosome-level genome assembly for the Pacific oyster Crassostrea gigas. Gigascience. 10(3):giab020. doi:10.1093/gigascience/giab020. 33764468 PMC7992393

[jkae138-B39] Regan T , Bean TP, Ellis T, Davie A, Carboni S, Migaud H, Houston RD. 2021. Genetic improvement technologies to support the sustainable growth of UK aquaculture. Reviews in Aquaculture. 13(4):1958–1985. doi:10.1111/raq.12553.

[jkae138-B40] Rhie A , Walenz BP, Koren S, Phillippy AM. 2020. Merqury: reference-free quality, completeness, and phasing assessment for genome assemblies. Genome Biol. 21(1):245. doi:10.1186/s13059-020-02134-9. 32928274 PMC7488777

[jkae138-B41] Ripabelli G , Sammarco ML, Grasso GM, Fanelli I, Caprioli A, Luzzi I. 1999. Occurrence of Vibrio and other pathogenic bacteria in Mytilus galloprovincialis (mussels) harvested from adriatic sea, Italy. Int J Food Microbiol. 49(1):43–48. doi:10.1016/S0168-1605(99)00056-2. 10477069

[jkae138-B42] Roach MJ , Schmidt SA, Borneman AR. 2018. Purge Haplotigs: allelic contig reassignment for third-gen diploid genome assemblies. BMC Bioinformatics. 19(1):460. doi:10.1186/s12859-018-2485-7. 30497373 PMC6267036

[jkae138-B43] Rodríguez-Juíz AM , Torrado M, Méndez J. 1996. Genome-size variation in bivalve molluscs determined by flow cytometry. Mar Biol. 126:489–497. doi:10.1007/BF00354631.

[jkae138-B44] Ruan J , Li H. 2020. Fast and accurate long-read assembly with wtdbg2. Nat Methods. 17(2):155–158. doi:10.1038/s41592-019-0669-3. 31819265 PMC7004874

[jkae138-B45] Saco A , Novoa B, Greco S, Gerdol M, Figueras A. 2023a. Bivalves present the largest and most diversified repertoire of toll-like receptors in the animal kingdom, suggesting broad-Spectrum pathogen recognition in marine waters. Mol Biol Evol. 40:6. doi:10.1093/molbev/msad133. PMC10279657 37279919

[jkae138-B46] Saco A , Rey-Campos M, Gallardo-Escárate C, Gerdol M, Novoa B, Figueras A. 2023b. Gene presence/absence variation in Mytilus galloprovincialis and its implications in gene expression and adaptation. iScience. 26(10):107827. doi:10.1016/j.isci.2023.107827. 37744033 PMC10514466

[jkae138-B47] Seuront L , Nicastro KR, Zardi GI, Goberville E. 2019. Decreased thermal tolerance under recurrent heat stress conditions explains summer mass mortality of the blue mussel Mytilus edulis. Sci Rep. 9(1):17498. doi:10.1038/s41598-019-53580-w. 31767954 PMC6877631

[jkae138-B48] Smit A , Hubley R, Green P. 2015. RepeatMasker Open-4.0. http://www.repeatmasker.org.

[jkae138-B49] Stanke M , Keller O, Gunduz I, Hayes A, Waack S, Morgenstern B. 2006. US: ab initio prediction of alternative transcripts. Nucleic Acids Res. 34(suppl_2):W435–W439. doi:10.1093/nar/gkl200. 16845043 PMC1538822

[jkae138-B50] Vaser R , Sović I, Nagarajan N, Šikić M. 2017. Fast and accurate de novo genome assembly from long uncorrected reads. Genome Res. 27(5):737–746. doi:10.1101/gr.214270.116. 28100585 PMC5411768

[jkae138-B51] Wang Y , Tang H, Debarry JD, Tan X, Li J, Wang X, Lee T-h, Jin H, Marler B, Guo H, et al 2012. MCScanx: a toolkit for detection and evolutionary analysis of gene synteny and collinearity. Nucleic Acids Res. 40(7):e49. doi:10.1093/nar/gkr1293. 22217600 PMC3326336

[jkae138-B52] Wilson J , Matejusova I, McIntosh RE, Carboni S, Bekaert M. 2018. New diagnostic SNP molecular markers for the Mytilus species complex. PLoS One. 13(7):e0200654. doi:10.1371/journal.pone.0200654. 30001394 PMC6042762

[jkae138-B53] Yang J-L , Feng D-D, Liu J, Xu J-K, Chen K, Li Y-F, Zhu Y-T, Liang X, Lu Y. 2021. Chromosome-level genome assembly of the hard-shelled mussel Mytilus coruscus, a widely distributed species from the temperate areas of east Asia. Gigascience. 10(4):giab024. doi:10.1093/gigascience/giab024. 33891010 PMC8063583

