# Supplementary material for: A chromosome-scale Mytilus edulis genome assembly for aquaculture, marine ecology, and evolution
Source: G3 (Bethesda). 2024 Jun 27;14(8):jkae138. doi: 10.1093/g3journal/jkae138 (PMC11304980; doi:10.1093/g3journal/jkae138)
Supplement: jkae138_Supplementary_Data [file jkae138_supplementary_data.docx]

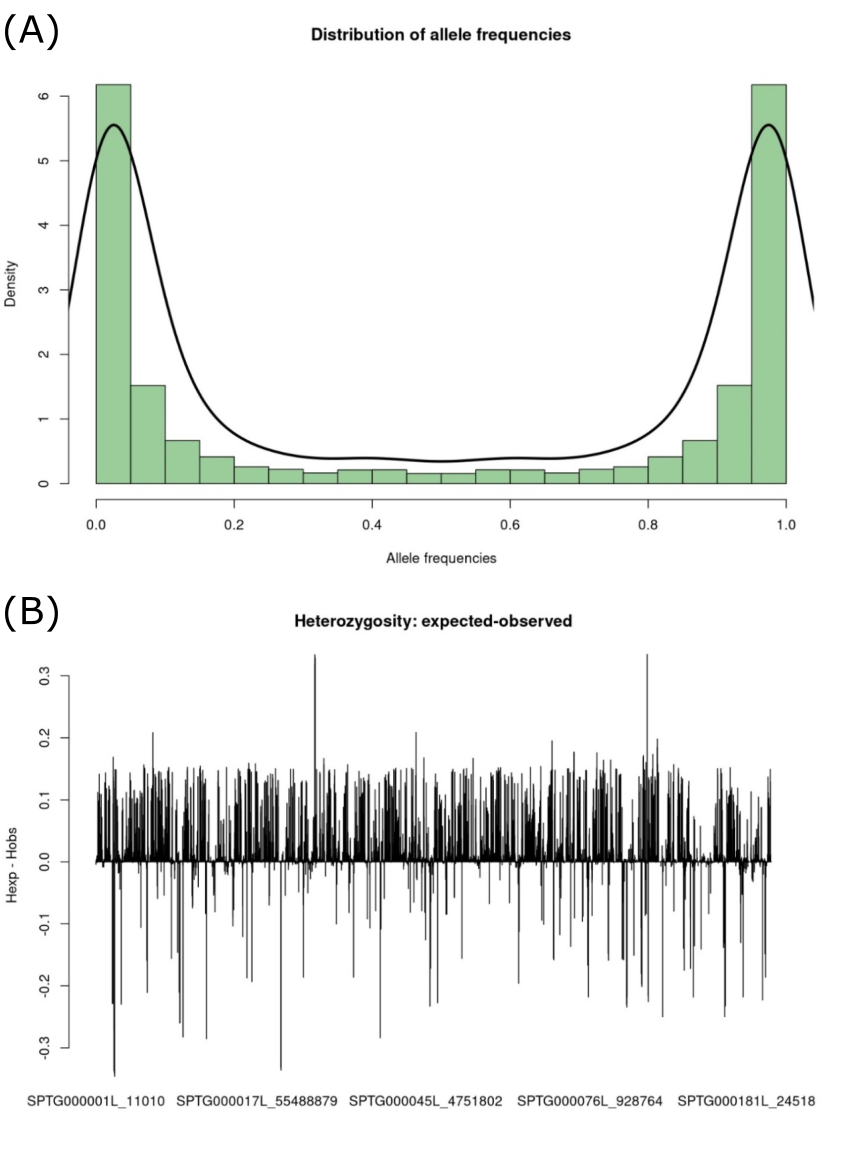


**Supplementary Figure 1: Allele frequency distribution for samples used in PCA.** (A) Allele frequencies are shown for each of the samples used in the population genetics analysis. (B) The deviation of observed from expected heterozygosity frequencies (y-axis) are shown for each of these samples along the genome (x-axis).

**Supplementary Table 1: Repeat Masker Summary Statistics**

| Total Sequences: | | | 1119 |  |  |
| --- | --- | --- | --- | --- | --- |
| Total length: | |  | 1651313236 bp |  |  |
| GC level: | |  | 32.30% |  |  |
| Bases masked: | | | 899369339 bp | (54.46%) |  |
|  |  |  |  |  |  |
|  |  |  | **No. of elements*** | **Length occupied (bp)** | **% of sequence** |
| **Retroelements** | | | **354908** | **144107995** | **8.73%** |
|  | SINEs: |  | 1372 | 268002 | 0.02% |
|  | Penelope: | | 0 | 0 | 0% |
|  | LINEs: |  | 312285 | 119718590 | 7.25% |
|  |  | *CRE/SLACS* | 0 | 0 | 0.00% |
|  |  | *L2/CR1/Rex* | 18764 | 9042646 | 0.55% |
|  |  | *R1/LOA/Jockey* | 4769 | 2238260 | 0.14% |
|  |  | *R2/R4/NeSL* | 318 | 445519 | 0.03% |
|  |  | *RTE/Bov-B* | 43204 | 17938137 | 1.09% |
|  |  | *L1/CIN4* | 1038 | 701049 | 0.04% |
|  | LTRements: | | 41251 | 24121403 | 0 |
|  |  | *BEL/Pao* | 6254 | 5338282 | 0.32% |
|  |  | *Ty1/Copia* | 2492 | 586513 | 0.04% |
|  |  | *Gypsy/DIRS1* | 21803 | 13956646 | 0.85% |
|  |  | *Retroviral* | 774 | 401029 | 0.02% |
| **DNA transposons** | | | **66456** | **26429217** | **1.60%** |
|  | hobo-Activator | | 5499 | 1548772 | 0.09% |
|  | Tc1-IS630-Pogo | | 7260 | 3955895 | 0.24% |
|  | En-Spm | | 0 | 0 | 0.00% |
|  | MULE-MuDR | | 6695 | 1566594 | 0.09% |
|  | PiggyBac | | 1519 | 258041 | 0.02% |
|  | Tourist/Harbinger | | 1837 | 578157 | 0.04% |
|  | Other (Mirage, P-element, Transib) | | 0 | 0 | 0.00% |
| Rolling-circles | | | 5920 | 3157017 | 0.19% |
| Unclassified: | |  | 2901690 | 712571709 | 43.15% |
| Total interspersed repeats: | | |  | 883108921 | 53.48% |
| Small RNA: | |  | 6827 | 1622139 | 0.10% |
| Satellites: | |  | 938 | 172825 | 0.01% |
| Simple repeats: | | | 203993 | 9365580 | 0.57% |
| Low complexity: | | | 40094 | 1942857 | 0.12% |
| * most repeats fragmented by insertions or deletions have been counted as one element | | | | | |


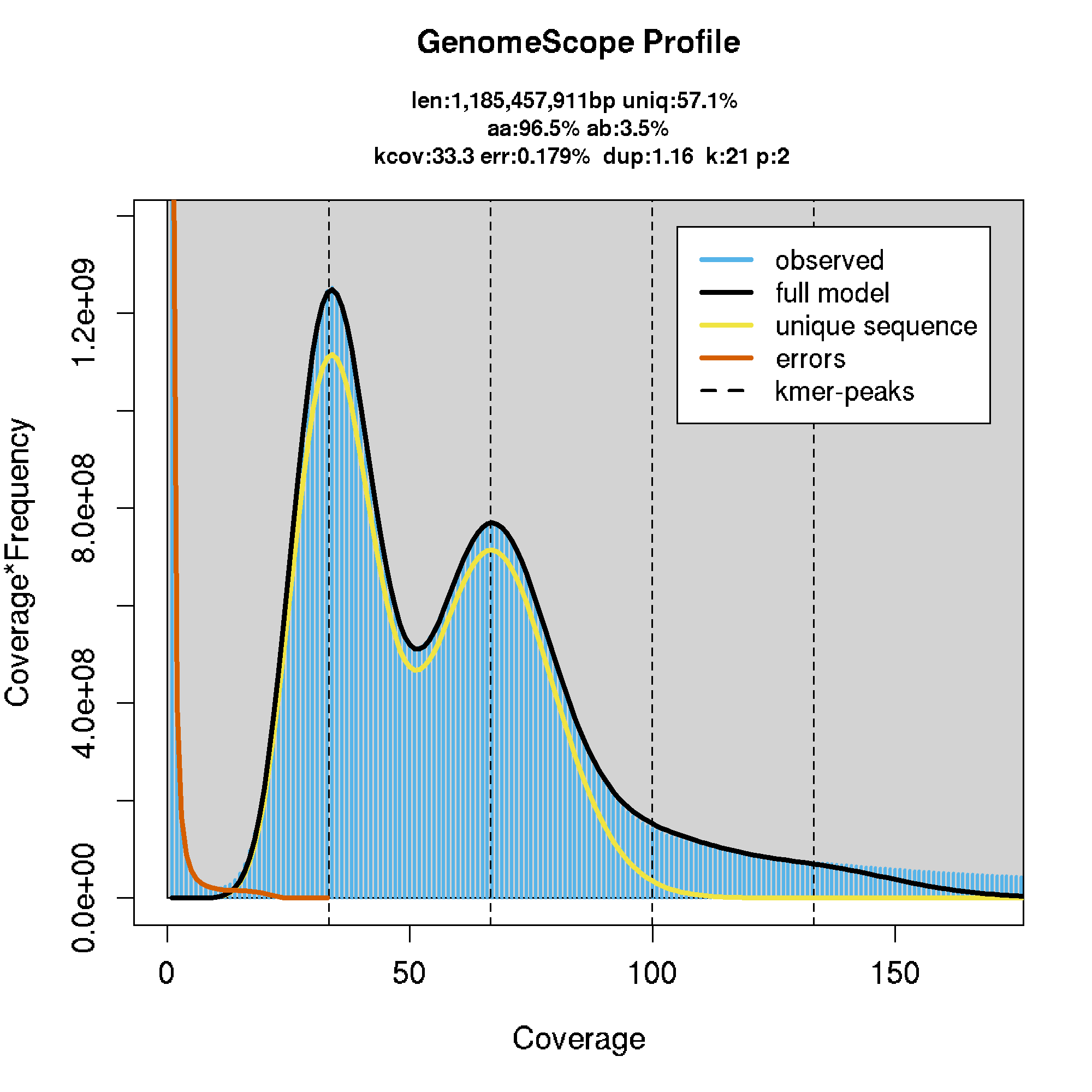


**Supplemental Figure 2: GenomeScope summary.** Shown are the summary statistics following analysis of the initial assembly using GenomeScope as described in the methods.
